# Iron Absorption from an Iron-Fortified Follow-Up Formula with and without the Addition of a Synbiotic or a Human-Identical Milk Oligosaccharide: A Randomized Crossover Stable Isotope Study in Young Thai Children

**DOI:** 10.1016/j.tjnut.2024.08.016

**Published:** 2024-08-22

**Authors:** Pornpimol Scheuchzer, Sangsom Sinawat, Anne-Sophie Donzé, Christophe Zeder, Magalie Sabatier, Marc Garcia-Garcera, Cristian Ricci, Thavatchai Kamontham, Michael B Zimmermann, Jeannine Baumgartner

**Affiliations:** 1 Laboratory of Human Nutrition, Institute of Food, Nutrition and Health, Department of Health Sciences and Technology, ETH Zurich, Zurich, Switzerland; 2 College of Allied Health Sciences, Suan Sunandha Rajabhat University, Bang Kaeo, Samut Songkhram, Thailand; 3 Nestlé Institute of Health Sciences, Nestlé Research, Société des Produits Nestlé S.A., Vers-Chez-Les-Blanc, Lausanne, Switzerland; 4 Africa Unit for Transdisciplinary Health Research, Faculty of Health Sciences, North-West University, Potchefstroom, South Africa; 5 Medical Research Council Translational Immune Discovery Unit, Weatherall Institute of Molecular Medicine, John Radcliffe Hospital, University of Oxford, Oxford, United Kingdom; 6 Department of Nutritional Sciences, Faculty of Life Sciences & Medicine, King’s College London, London, United Kingdom

**Keywords:** follow-up formula, galacto-oligosaccharides, iron absorption, *Limosilactobacillus reuteri* (*L. reuteri*), stable isotope, synbiotic, 2′-fucosyllactose, young children, Thailand

## Abstract

**Background:**

Previous studies showed that pre- and probiotics may enhance iron absorption. Probiotics combined with prebiotics (synbiotics), including human-identical milk oligosaccharides (HiMOs), are commonly added to infant and follow-up formula (FUF). Whether these additions enhance iron absorption from iron-fortified commercial milk formula is uncertain.

**Objectives:**

We determined the effect of adding *1*) a synbiotic [galacto-oligosaccharide [GOS] + *Limosilactobacillus reuteri* (*L. reuteri*)] or *2*) the HiMO 2′-fucosyllactose (2′FL) to iron-fortified FUF on iron absorption in young Thai children.

**Methods:**

In a randomized, controlled, single-blinded (participants) crossover study, 82 Thai children aged 8–14 mo were enrolled to consume single servings (235 mL) of FUF with isotopically labeled ferrous sulfate (2.2 mg iron) with *1*) the synbiotic (400 mg/100 mL GOS and *L. reuteri* DSM 17938), *2*) the HiMO 2′FL (100 mg/100 mL), and *3*) without synbiotic and 2′FL (control) in random order and a 3-d washout period between administrations. Fractional iron absorption [FIA (%)] was assessed by measuring erythrocyte incorporation of isotopic labels 14 d (*n* = 26) and 28 d (*n* = 76) after consumption of the last test FUF.

**Results:**

Median (IQR) FIA from iron-fortified FUF with the synbiotic [8.2 (5.2, 12.9)%] and with 2′FL [8.4 (5.5, 14.1)%] did not differ from the control FUF [8.1 (4.8,14.7)%] (synbiotic compared with control, *P* = 0.24; 2′FL compared with control, *P* = 0.95). FIA from all FUF did not differ when measured after 14 and 28 d of erythrocyte incorporation (Time, *P* = 0.368; FUF, *P* = 0.435; Time × FUF, *P* = 0.937). Fecal pH and hemoglobin were negatively associated with FIA.

**Conclusions:**

In young Thai children, the addition of a synbiotic (GOS + *L. reuteri*) or 2′FL to iron-fortified FUF did not impact FIA from a single serving.

The study was registered at clinicaltrials.gov as NCT04774016.

## Introduction

Globally, iron deficiency anemia (IDA) is among the 5 leading causes of years lived with disability [1]. Infants and young children in low- and middle-income countries are at particular risk because their iron intakes are often insufficient to meet increased requirements for rapid growth and development [2].

The WHO recommends exclusive breastfeeding for the first 6 mo of life, followed by the introduction of complementary foods at 6 mo together with continued breastfeeding for ≤2 y and beyond [3]. Around 6 mo of age, the iron content in mature human milk (∼0.3 mg/L) alone is no longer sufficient to meet physiologic requirements, and therefore additional sources of iron need to be introduced during complementary feeding [4]. Infants who are fed kinds of milk other than human milk are recommended by WHO to be fed animal milk once they reach 12 mo of age [5].

Prebiotic galacto-oligosaccharides (GOSs) alone or in combination with probiotic bacteria (synbiotics) are commonly added to commercial milk formula in an attempt to mimic the natural prebiotics and probiotic bacteria present in human milk [6,7]. Whether these additions enhance iron absorption from nonheme iron-fortified commercial milk formula is uncertain.

In a previous stable iron isotope study in mostly iron-deficient anemic Kenyan infants, cofortification of maize porridge with iron and prebiotic GOSs in a micronutrient powder increased iron absorption by 62% compared with iron fortification alone [8]. Moreover, GOS mitigated the adverse gastrointestinal (GI) side effects caused by unabsorbed fortification iron by selectively enhancing the growth of beneficial colonic bacteria [9,10].

*Limosilactobacillus reuteri* (*L. reuteri;* formerly *Lactobacillus reuteri*) is a well-studied probiotic bacterium found in various parts of the human body, including the GI tract and human milk [11]. The impact of *L. reuteri* on iron absorption has, to our knowledge, not been studied. However, previous iron isotope studies in Swedish females of varying iron status reported an increase in iron absorption from a fruit drink and test meals when supplemented with *Lactobacillus plantarum* [[12], [13], [14]].

Human milk oligosaccharides (HMOs) are linear and branched structure carbohydrates naturally present in human milk [15], with >200 oligosaccharide structures identified to date [16]. In the milk of the majority of females, 2′-fucosyllactose (2′FL) is the most abundant HMO [17]. Supplementing infant formula with the human-identical oligosaccharide (HiMO) 2′FL is considered safe, but clinical studies on the health benefits in infants and young children are currently limited [[18], [19], [20], [21]]. A recent study in partially breastfed Kenyan infants showed that the addition of the HiMOs 2′FL and lacto-N-neotetraose to iron-fortified maize porridge does not affect iron absorption [22]. Whether HiMOs affect iron absorption from commercial milk formula, including infant formula and follow-up formula (FUF), has to our knowledge not been investigated.

Therefore, our co-primary objectives were to determine the effect of adding *1*) a synbiotic (GOS + *L. reuteri* DSM 17938) and *2*) 2′FL to iron-fortified FUF on iron absorption in young Thai children.

We hypothesized that supplementing FUF with a synbiotic containing a mixture of GOS and *L. reuteri* or 2′FL would increase iron absorption from FUF fortified with iron as ferrous sulfate (FeSO_4_). FeSO_4_ is the iron compound of choice for iron fortification of infant formula due to its high solubility and therefore high bioavailability [23].

We commonly assess fractional iron absorption (FIA) in adults and children by measuring erythrocyte incorporation of iron isotopic labels 14 d after administration of a test condition labeled with stable iron isotopes. A previous study by Fomon et al. [24] suggested that in infants incorporation of isotopic labels into erythrocytes continues until 28 d after administration. Thus, in the current study, we measured erythrocyte incorporation 28 d after the last test FUF consumption, and after 14 d in a subsample (*n* = 26) for comparison.

## Methods

### Study site and participants

The study was conducted at Amphawa Hospital in the Samut Songkhram province of central Thailand between November 2020 and March 2021. We recruited caregiver–child pairs from 47 primary healthcare centers in the Samut Songkhram province. Inclusion criteria for the children were *1*) age 8–14 mo, *2*) no clinical signs or symptoms of chronic disease or acute illness, *3*) capillary hemoglobin (Hb) >70 g/L, *4*) not severely underweight or wasted [weight-for-age (WAZ) and weight-for-length (WLZ) Z-scores >−3], *5*) singleton, full-term birth (≥37 weeks), *6*) birth weight between ≥2.5 kg and ≤4.5 kg, and *7*) anticipated residence in the area for the study duration. Children were excluded if they: *1*) received vitamin and mineral supplements in the 2 weeks before enrollment, *2*) received all milk feeds in the form of human milk and were not receiving infant or FUF at the time of screening, *3*) received antibiotic treatments in the 4 weeks before enrollment, and *4*) were allergic or intolerant to cow milk protein or lactose or had severe food allergies. The protocol registered on clinicaltrials.gov states that children aged 10–14 mo will be recruited, but we widened the age range to speed up recruitment and study completion due to the ongoing COVID-19 pandemic.

### Ethical considerations

The ethical committees of ETH Zurich (EK 2020-N-33), the College of Allied Health Sciences at Suan Sunandha Rajabhat University (COA 1-005/2020), and the Ministry of Health (COA No 16/2563) of Samut Songkhram province approved the study. It was carried out in accordance with principles enunciated in the current version of the Declaration of Helsinki, the guidelines of Good Clinical Practice issued by the International Council on Harmonization, as well as legal regulations in Switzerland and Thailand. We obtained written informed consent from all mothers or court-consent guardians before screening and enrollment. This trial was registered at clinicaltrials.gov as NCT04774016.

### Study design

**FIGURE 1:**
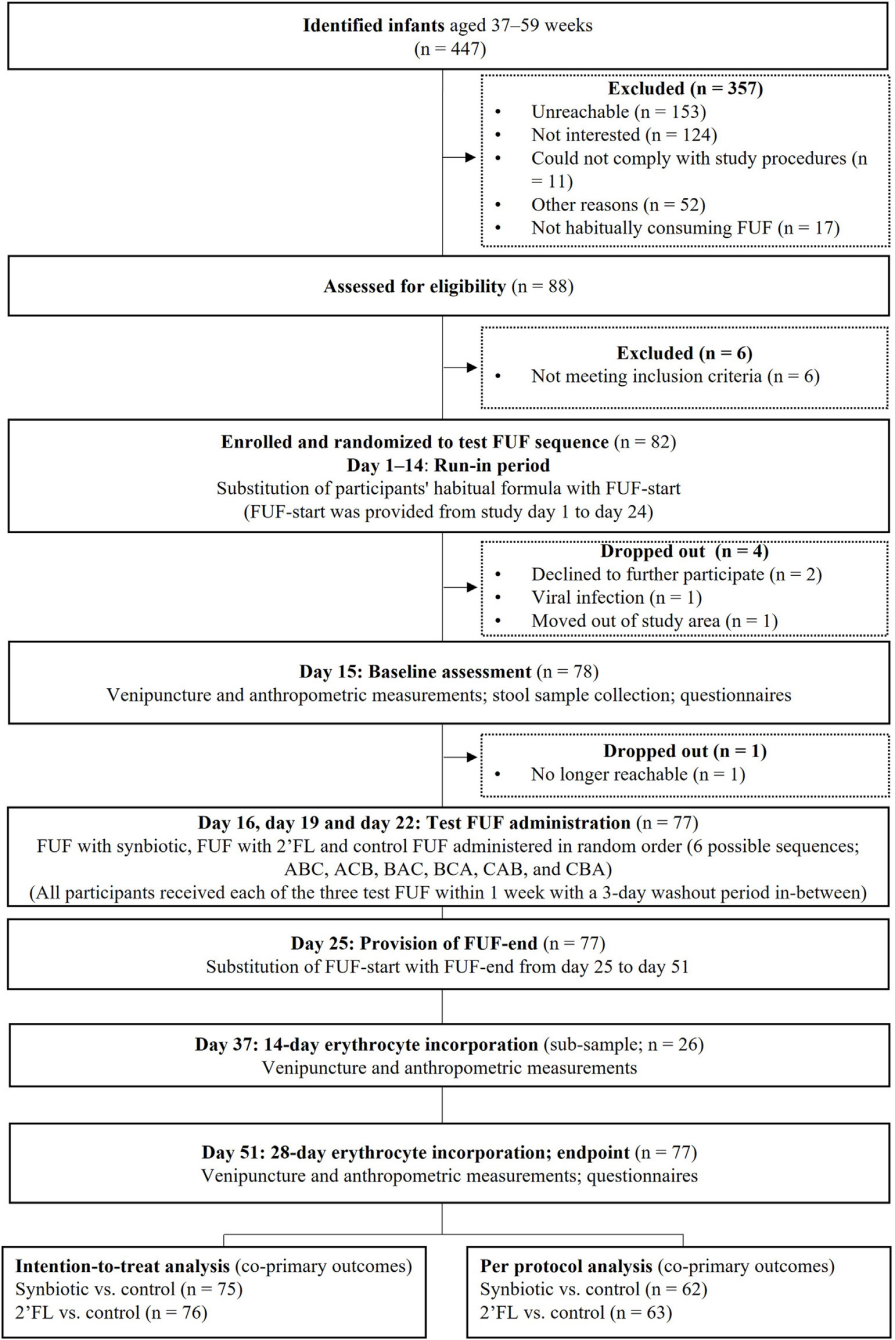
Study design and participant flowchart. FUF, follow-up formula; 2′FL, 2′-fucosyllactose.

This study was a randomized, controlled, single-blind (participants), crossover trial (Figure 1). The primary outcome was FIA from single servings (235 mL) of FUF fortified with isotopically labeled FeSO_4_ with addition of *1*) a synbiotic (400 mg/100 mL GOS and *L. reuteri* DSM 17938 at a concentration that guarantees ∼10^8^ colony-forming units (cfu)/d; ^57^FeSO_4_), *2*) 2′FL (100 mg/100 mL; ^54^FeSO_4_), and *3*) without synbiotic or 2′FL (^58^FeSO_4_) as control. The HiMO tested in the study was obtained from fermentation process. All 3 test FUF contained 2.20 mg of the respective labeled iron source and 0.05 mg iron as native iron per 235 mL serving and were consumed by the children in random order. FIA was assessed by erythrocyte incorporation of isotopic labels 28 after FUF consumption, as well as after 14 d in a subsample (subsample not defined in clinicaltrials.gov). During the incorporation period, each child consumed the FUF with synbiotics in place of their habitual formula. In this article, we are not addressing the objectives of a nested before-after study stipulated in clinicaltrials.gov.

### Study procedures

Caregiver–child pairs were recruited from primary health care clinics by the study manager (pediatrician) and trained study assistants [BSc public health and allied health sciences students and the PhD in human nutrition student (PS)]. Only caregivers of children who were fed with infant formula or FUF were given more detailed information about the study. Caregiver–child pairs who expressed interest in participating in the study after receiving the study information (verbally and in writing) were invited to attend a screening visit at the Amphawa hospital. At screening, after caregivers gave informed consent, we measured the child’s Hb concentrations in a capillary blood spot obtained by finger prick. We further collected information on eligibility, caregiver–child demographics, and feeding practices (history and current) in a structured interview, and the study pediatrician assessed the children’s health status including anthropometric parameters. Children who fulfilled the inclusion and exclusion criteria were enrolled in the study and randomized (without stratification) to 1 of 6 test FUF sequences (ABC, ACB, BAC, BCA, CAB, and CBA) using a dynamic allocation algorithm from Medidata Balance.

The total study duration was 51 d (Figure 1). Caregivers were asked (verbally) by a trained study assistant to substitute their child’s habitual formula with a FUF (washout FUF) that was similar in composition to the test FUF, but lower in iron content (1.9 mg/235 mL serving) and without the addition of any pre- or probiotics (Supplemental Table 1) during a 14-d run-in period (day 1–14) and until study day 25. Caregivers were encouraged to feed their children 2 servings (∼235 mL/serving) of this washout FUF per day but without an attempt to force compliance. They were asked not to replace habitual breastmilk feeds in children who received human milk in addition to formula. The purpose of the 14-d run-in period was to ensure washout of pre- or probiotics in the GI tract from the participating children’s habitual formula, which all contained a form of pre- or probiotics. We further instructed caregivers to collect stool samples from their child at home on day 14 into an OMNIgene GUT OM-200 (DNAgenotek) kit and a screw-cap polypropylene stool container.

On day 15, the caregiver–child pairs visited the study site for baseline data collection, which entailed the withdrawal of blood by venipuncture for baseline iron isotopic analysis, and measurement of Hb, iron status [plasma ferritin (PF), soluble transferrin receptor (sTfR)] and inflammation biomarkers [C-reactive protein (CRP) and alpha-1 acid glycoprotein (AGP)], as well as intestinal fatty acid binding protein (I-FABP), a biomarker of gut integrity. We collected stool samples to analyze fecal pH as a potential factor affecting iron absorption from the distal intestine. On days 16, 19, and 22, the respective isotopically labeled test FUF (235 mL; Supplemental Table 1) was administered between 6:30 a.m. to 7:30 a.m. to the children under close supervision. Caregivers were requested not to feed food other than the provided washout FUF without added pre- and probiotics or human milk during the night before the visit, and to keep the children fasting for 3 h before the scheduled appointment at the study site. The respective test FUF had to be consumed in full and the bottle/cup was rinsed 3 times with 10 mL water (30 mL of water in total). After test FUF consumption, the caregiver–child pairs were requested to stay at the study site for 2 h, during which the child was encouraged not to eat or drink and observed for any spit-up or vomited FUF. Breastfed children were able to feed on demand during this period, which was noted by a study team member for consideration in statistical analysis. The 3 isotopically labeled test FUF administration visits were separated by a 3-d washout period, during which the caregivers were asked to substitute all habitual formula feeds with the washout FUF without added pre- and probiotics and low in iron.

Three days after consumption of the last isotopically labeled test FUF (on day 25), caregivers were asked to substitute all habitual formula feeds with a FUF containing 2.20 mg FeSO_4_ per serving (235 mL) and the synbiotic (GOS + *L. reuteri* DSM 17938) (Supplemental Table 1), until day 51 (study endpoint). On day 51, 28 d after the last FUF administration, we collected another venous blood sample to assess erythrocyte incorporation of isotopic labels to determine FIA from the 3 different test FUF. In a subsample of volunteering caregiver–child pairs, we collected an additional venous blood sample on day 37 to assess erythrocyte incorporation of isotopic labels 14 d after the last test FUF administration.

At screening, days 15, 37, and 51, we measured the child's weight using a Salter-type infant weighing scale to the nearest 0.1 kg and assessed the child's length using a rigid measurement board to the nearest 0.5 cm. We calculated WAZ, WLZ, and length-for-age Z-scores using WHO Anthro version 3.2.2 (WHO). Adverse events (AE) and serious adverse events (SAE) were assessed at each visit by the study pediatrician and were reported to and reviewed by the Data and Safety Monitoring Board and the 2 Ethics Committees. Participants received reimbursement for travel costs and compensation for their time (a total of 500 Thai Baht per visit). At the last study visit, the trained research assistants provided caregivers with advice on optimal child feeding practices aligned with the information in the mother and child health handbook (“the Pink Book”).

### Stable isotope labels

We prepared 2.2 mg doses of ^58^Fe-, ^57^Fe-, and ^54^Fe-labeled FeSO_4_ from ^58^Fe, ^57^Fe, and ^54^Fe-enriched elemental iron (99.90%, 95.6%, and 99.4% isotopic enrichment, respectively; Chemgas, Boulogne, France) by Dr Paul Lohmann GmbH. The ^58^FeSO_4_, ^57^FeSO_4_, and ^54^FeSO_4_ solutions were prepared by dissolution in 0.1 mol/L sulfuric acid to reach a concentration of 1.1 mg Fe/mL. Labeled iron compounds were analyzed for iron isotopic composition and the tracer iron concentration via isotope-dilution mass spectrometry as outlined below. These labeled FeSO_4_ solutions were kept at 4˚C until further use for the preparation of isotopically labeled test FUF.

### Composition and preparation of follow-up formulas

All FUF used in this study were produced according to the specifications for a commercial product at the Nestlé Product Technology Center. The nutritional composition of the different FUF is presented in Supplemental Table 1 (key characteristics). All FUF had an osmolality of 342 mOsm/kg. We prepared the isotopically labeled test FUF servings by mixing 32.3 g of formula powder with 210 mL water (boiled and cooled to 40°C) in the feeding bottle/cup. For the FUF containing 2′FL, we mixed formula powder with 210 mL 2′FL solution (1 g/L) from a tetra pack warmed to 40°C. We then added 2 mL isotopically labeled ^58^FeSO_4_, ^57^FeSO_4_, and ^54^FeSO_4_ solution (2.2 mg iron) to the feeding bottle containing FUF supplemented with synbiotic (GOS and *L. reuteri* DSM 17938), 2′FL, and no synbiotic or 2′FL (control), respectively, and immediately fed it to the children.

The washout FUF included 1.9 mg iron per 235 mL serving and no added pre- or probiotics. The nutritional composition was otherwise identical to the test FUF. The FUF provided during the incorporation period was identical to the test FUF containing the synbiotic, except that the FeSO_4_ was not labeled. The respective formula powder was provided to the caregiver–child pairs in 400 g cans. The number of cans provided to each caregiver was aligned with their reported habitual formula use at the screening visit. Trained study assistants (BSc Public Health and Allied Health Sciences students) instructed caregivers verbally on how to reconstitute the FUF according to the instructions provided on the label. All FUF were provided free of charge. We further provided feeding equipment (bottle, cup, and bib) to participants.

### Laboratory analyses

#### Blood analysis

We measured Hb using a HemoCue 301 analyzer (Angelholm, Sweden), calibrated daily using Hb301 standard solution (Eurotrol), in capillary whole blood at screening and venous whole blood at days 15, 37, and 51. The study site was almost at sea level. Thus, no Hb adjustment for altitude was required. Venous blood samples (2 mL) were drawn into 4 mL heparin-coated tubes (BD, Germany) on days 15, 37, and 51. Aliquots of whole blood, and plasma obtained by centrifuging the remaining blood for 10 min at 1500 × *g*, were stored at −20°C and shipped frozen to ETH Zurich, Switzerland, for analysis.

We measured biomarkers of iron status (PF and sTfR) and inflammation (AGP and CRP) in plasma using a multiplex immunoassay [25]. We adjusted PF for inflammation using the Biomarkers Reflecting Inflammation and Nutritional Determinants of Anemia regression correction approach [26]. Body iron stores were estimated from the ratio of sTfR to PF [27]. Anemia was defined as Hb <110 g/L [28]. Iron deficiency was defined as PF <12 μg/L [29] and/or elevated sTfR >8.3 mg/L [25]. IDA was defined as the combination of anemia and iron deficiency. CRP and AGP values >5 mg/L and >1 g/L, respectively, were described as indicating inflammation [25]. We measured I-FABP in plasma using a commercially available ELISA kit (Hycult Biotech).

Whole blood samples collected on days 15, 37, and 51 were mineralized in duplicate by microwave-assisted digestion in nitric acid (TurboWave, MLS) followed by separation of the iron from the blood matrix via anion-exchange chromatography and a subsequent precipitation step with ammonium hydroxide as described previously [8,30]. We measured isotope ratios using inductively coupled plasma mass spectrometry (Neptune, Thermo Finnigan) equipped with a multicollector system for simultaneous iron beam detection [30].

#### Calculation of iron absorption

We calculated the amounts of ^58^Fe, ^57^Fe, and ^54^Fe isotopic labels in whole blood 14 and 28 d after administration of the last test FUF based on the shift in iron isotopic ratios and the estimated amount of iron circulating in the body. Circulating iron was calculated based on Hb concentrations and blood volume estimated from weight, length, and measured Hb at the time of blood collection [31]. The calculation was based on principles of isotope dilution and took into account that iron isotopic labels are not monoisotopic, using the methods described by Turnlund et al*.* [32] and Cercamondi et al. [33]. For the calculation of FIA, we assumed a 75% incorporation of the absorbed iron based on a previous study that measured erythrocyte isotope incorporation from intravenously infused ^58^Fe in Ghanaian children [34].

#### Fecal analysis

We measured fecal pH, a total of 100 mg (±10%) of feces was added to 1 mL ultrapure water (>18 mΩ-cm), vortex for 30 s and centrifuged for 3 min at 5000 × *g* at 4°C. The pH in the liquid phase was measured with a digital pH meter (Metrohm).

### Sample size calculation

The sample size was calculated for the detection of a 20% within-subject difference in FIA from FUF with synbiotic or 2′FL and the control FUF. Based on a within-subject standard deviation of 0.206 from log-10 transformed (0.474 on the natural-log scale) iron absorption in a previous study performed in Kenyan infants [4], an experiment-wise false positive rate of 5% and a power of 90%, the estimated sample size was 74 subjects. To compensate for 10% dropouts, we enrolled 82 children.

### Data and statistical analysis

Statistical analyses were performed using SAS (Version 9.4) and IBM SPSS-26 (version 26; SPSS). We evaluated the normality of data using Q–Q plots and histograms. Normally distributed data are presented in the text, tables, and figures as mean ± SD, not normally distributed data as median and IQR, and categorical data as frequencies and percentages [*n* (%)].

We compared (log-transformed) FIA from the different iron-fortified FUF within subjects using mixed models with formula as fixed effect and subject as a random factor. We also ran a sensitivity analysis including a sequence as fixed effect. Of interest were the 2 primary comparisons: synbiotic compared with control and 2′FL compared with control. The experiment-wise error rate of these 2 comparisons was controlled according to Bonferroni-Holm. Stratification by potential variables of interest (for example, iron status and fecal pH) coded as dichotomous variables according to baseline median values or clinically relevant thresholds was conducted. We further explored factors associated with FIA from the different test FUF using multiple linear regression analysis. Variables included in the enter models were Hb, PF, sTfR, CRP, age, and fecal pH. In a subsample, we further compared (log-transformed) FIA from the different iron-fortified FUF measured 14 d compared with 28 d after the last test FUF consumption using mixed models with formula and time as fixed effect and subject as a random factor. Linear model assumptions were investigated looking at normality of residuals and absence of outliers by visual inspection of the histogram, Q–Q plots, and by the plot of residuals compared with fitted values. The homoscedasticity assumption was investigated using the Breush pagan test. Two-sided *P* values of <0.05 were considered statistically significant.

## Results

### Participants

We identified 447 children through the primary health centers and invited 88 interested caregiver–child pairs for screening (Figure 1). We enrolled 82 children; 4 dropped out during the 14-d run-in period for the following reasons: declined to further participate (*n* = 2), viral infection (*n* = 1), moved out of the study area (*n* = 1), and 1 was no longer reachable after baseline assessment (*n* = 1). Subsequently, 77 children attempted to consume the test FUF and completed the study.

Randomization to FUF administration sequence was balanced with *n* = 11–14 participants in each of the 6 sequence groups. For the primary outcomes, we performed both an intention-to-treat (ITT) and a per-protocol (PP) analysis. For the ITT analysis, we excluded data of 1 child who received the same isotope twice. One child further did not consume FUF with synbiotics and, therefore, could not be included in the synbiotic compared with the control FUF comparison. Because subjects were their own controls, this resulted in *n* = 75 for the synbiotic compared with the control comparison and *n* = 76 for the 2′FL compared with control comparison. For the PP analysis, we additionally excluded data of children who *1*) had signs of inflammation/infection (CRP >5 mg/L) at baseline (*n* = 4), *2*) consumed <50% of the administered test FUF volume due to preferring to drink breastmilk (*n* = 5, control; *n* = 4 synbiotic; *n* = 2, 2′FL), *3*) vomited within 2 h of test FUF consumption (*n* = 1 synbiotic; *n* = 2, 2′FL), or *4*) fasted <2 h before administration (*n* = 1, control). This resulted in *n* = 63 for the 2′FL compared with the control comparison, and *n* = 62 for the synbiotic compared with the control comparison.

Baseline characteristics of the children are shown for the ITT sample (*n* = 76) in Table 1 and for the PP sample (*n* = 63) in Supplemental Table 2. The mean ± SD age of the children was 50.9 ± 7.0 wk and 55% were female. Among the 76 children, 12 (16 %) were anemic, 16 (23%) were iron deficient (adjusted PF <12 μg/L and/or sTfR >8.3 mg/L), and 5 (7%) had IDA. Four (5%) and 7 (6%) children had elevated CRP (>5 mg/L) and AGP (>1 mg/L), respectively. Twenty-one (28%) children were partially breastfed at baseline and 20 (26%) remained partially breastfed at endpoint. Median (IQR) reported habitual formula intake was 840 (540, 1275) mL/d, with no increase from baseline to endpoint [840 (630, 1245); *P* = 0.348].

**TABLE 1:** Baseline characteristics of the 8–14 mo-old Thai children participating in the stable iron isotope absorption study.

Characteristic	*n*	Value
Age (wk)	76	50.9 ± 7.0^1^
Male/female, *n* (%)	76	34/42 (45/55)
Age at formula introduction (mo)	76	2 (0, 5)
Receiving human milk during study, *n* (%)	76	21 (28)
Reported habitual formula intake (mL/d)	76	840 (540, 1275)
Habitual formula, *n* (%)	76	
Soya formula		2 (3)
Infant formula		21 (28)
Follow-up formula		39 (51)
Growing-up milk		14 (18)
Anthropometrics		
Weight (kg)	76	9.2 ± 1.2
Length (cm)	76	74.0 ± 3.1
Weight-for-age z-score	76	−0.0 ± 1.0
Weight-for-length z-score	76	0.10 ± 1.1
Length-for-age z-score	76	−0.2 ± 1.0
Iron status		
Hemoglobin (g/)L	75	122 ± 10
Anemia, *n* (%)^3^	75	11 (15)
Plasma ferritin (μg/L)	71	30.3 (19.9, 49.3)^2^
Plasma ferritin adjusted^4^ (μg/L)	71	26.7 (17.6, 45.6)
<12 μg/L, *n* (%)	71	8 (11)
Soluble transferrin receptor (mg/L)	71	6.3 (5.3, 7.3)
>8.3 (mg/L), *n* (%)	71	12 (17)
Body iron stores (mg/kg body weight)	71	4.5 (2.4, 5.8)
Iron deficiency, *n* (%)^5^	71	16 (23)
Iron deficiency anemia^6^, *n* (%)	71	5 (7)
Systemic inflammation		
C-reactive protein, mg/L	71	0.1 (0.0, 0.7)
>5 (mg/L), *n* (%)	71	4 (6)
α-1-acid glycoprotein (g/L)	71	0.6 (0.5, 0.8)
>1 (mg/L), *n* (%)	71	7 (10)

Abbreviations: BRINDA, Biomarkers Reflecting Inflammation and Nutritional Determinants of Anemia; Hb, hemoglobin; PF, plasma ferritin; sTfR, soluble transferrin receptor.

1 Mean ± SD, all such values.

2 Median (IQR), all such values.

3 Anemia defined as Hb <110 g/L.

4 Plasma ferritin adjusted for inflammation by BRINDA.

5 Iron deficiency was defined as adjusted PF <12 μg/L and/or elevated sTfR >8.3 mg/L.

6 Iron deficiency anemia is defined as the combination of anemia and iron deficiency.

Seasonal influenza accounted for ∼75% (*n* = 33) of the total AE cases reported during the trial, followed by diarrhea of ∼10% (*n* = 6) and other causes of ∼10% (*n* = 6) such as insect bites, rashes, and skin injuries due to falling. SAE occurred in 2 cases (*n* = 2) due to gastroenteritis and herpangina. AE and SAE were closely monitored by the study pediatrician and all cases were resolved.

### Iron absorption

**FIGURE 2:**
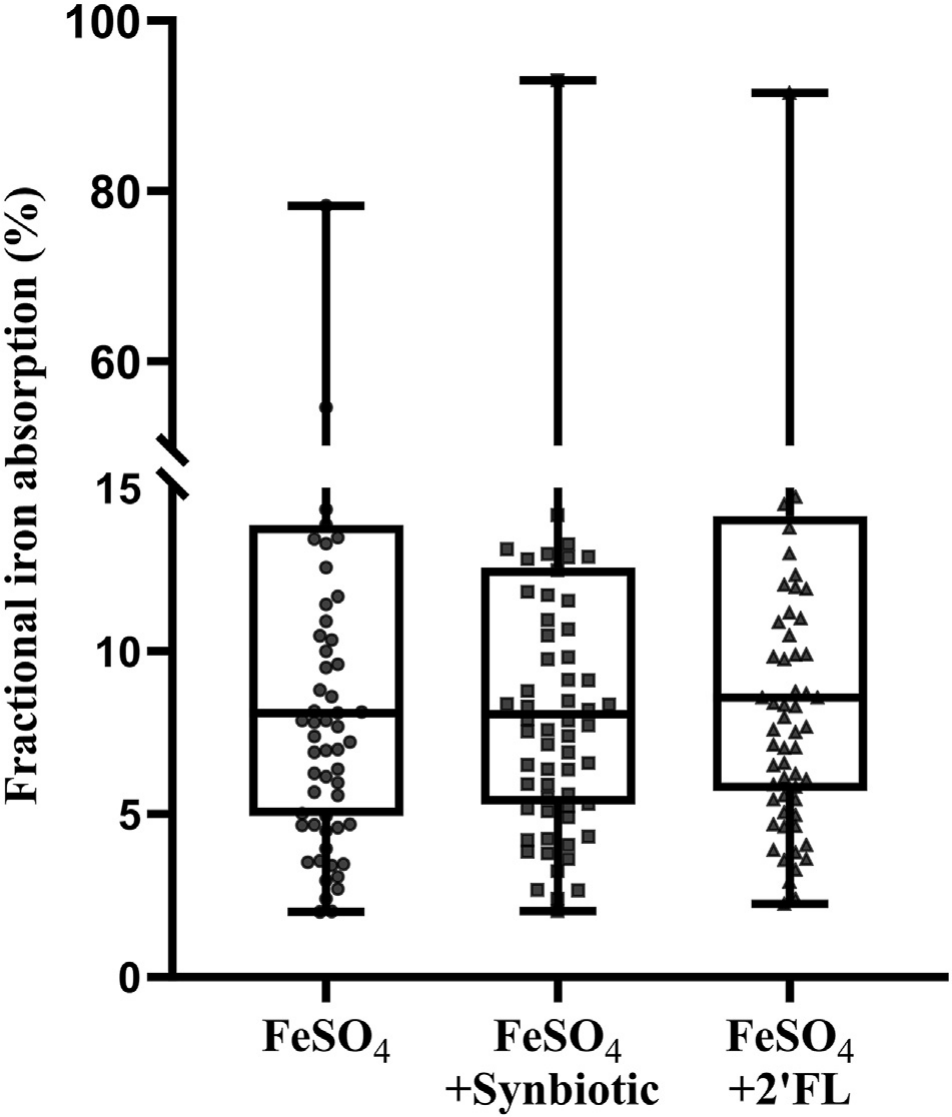
Fractional iron absorption (FIA) (%) in young Thai children from follow-up formulas (FUF) containing 0.5 mg native iron and 2.20 mg isotopically labeled iron and *1*) synbiotic (galacto-oligosaccharides + *L. reuteri*) (*n* = 75), *2*) 2′FL (*n* = 76), and *3*) without synbiotic or 2′FL (control; *n* = 76). The presented data are from the intention-to-treat analysis. We compared (log-transformed) FIA from the different iron-fortified FUF within subjects using mixed models with formula as fixed effect and subject as a random factor. There were no differences in FIA between the FUF with added synbiotics or 2′FL and the control FUF (*P* > 0.05, both comparisons). The boxes show the medians with 25th and 75th percentiles, and the dots represent individual values. FeSO_4_, ferrous sulfate; 2′FL, 2′-fucosyllactose.

Median (IQR) FIA (%) from the 3 isotopically labeled FUF in the ITT sample are shown in Figure 2. FIA from iron-fortified FUF with synbiotic [8.2 (5.2, 12.9) %] and with 2′FL [8.4 (5.5, 14.1) %] did not differ from the control FUF [8.1 (4.8, 14.7) %] (synbiotic compared with control, *P* = 0.24; 2′FL compared with control, *P* = 0.95). We also found no differences in FIA from iron-fortified FUF supplemented with synbiotic [8.2 (5.3, 12.8) %] or 2′FL [8.6 (5.8, 14.5) %] when compared with control FUF [8.1 (5.0, 13.8) %] in the PP analysis (synbiotic compared with control, *P* = 0.28; 2′FL compared with control, *P* = 0.57). Adjusting for FUF administration sequence did not affect the results (synbiotic compared with control, *P* = 0.70; sequence, *P* = 0.34; sequence × FUF, *P* = 0.86 and 2′FL compared with control, *P* = 0.61; sequence, *P* = 0.23; sequence × FUF, *P* = 0.59).

**FIGURE 3:**
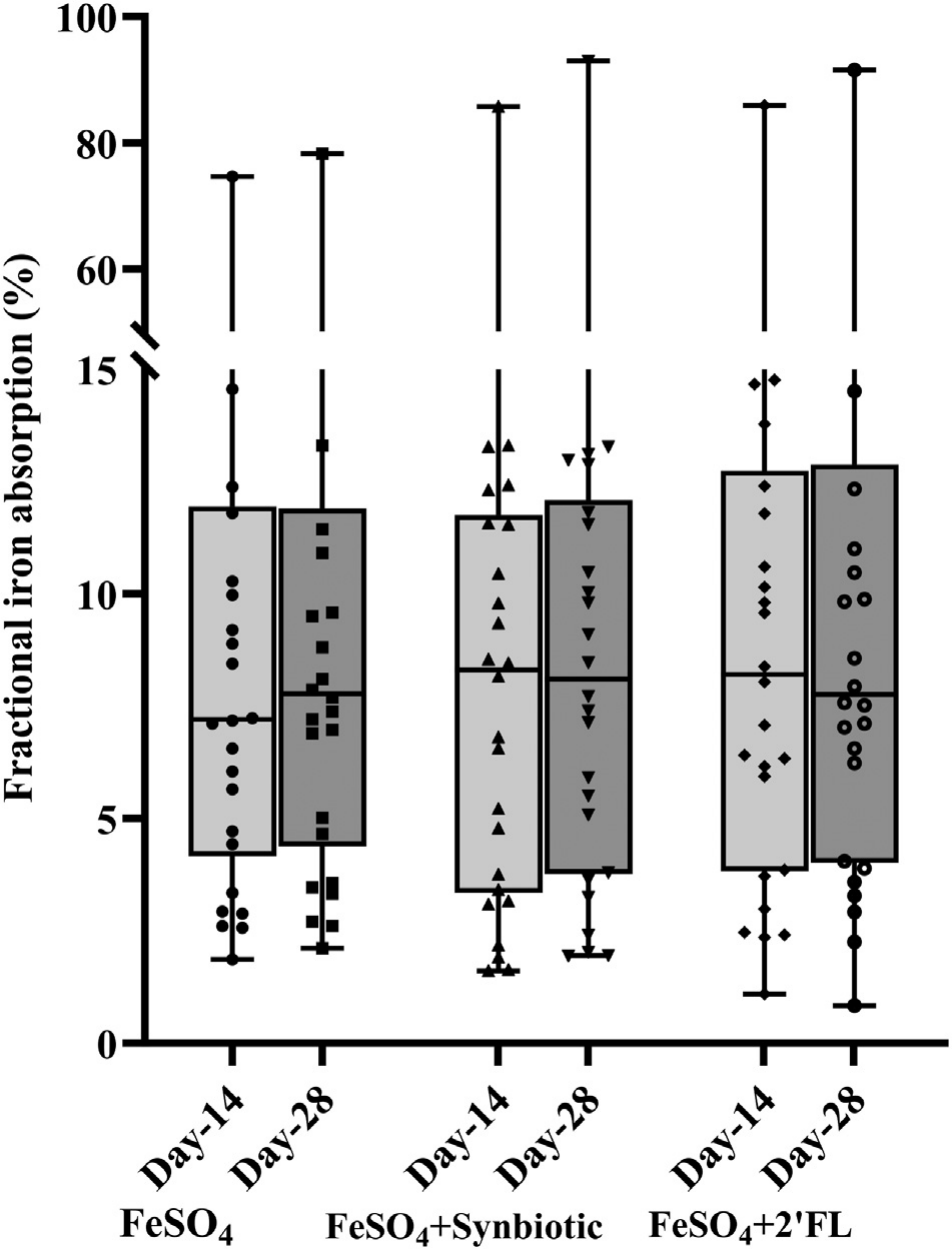
Fractional iron absorption (FIA) (%) in young Thai children from follow-up formulas (FUF) containing 0.5 mg native iron and 2.20 mg isotopically labeled iron and *1*) synbiotic (galacto-oligosaccharides + *L. reuteri*) (*n* = 26), *2*) 2′FL (*n* = 26), and *3*) without synbiotic or 2′FL (control; *n* = 26) measured after a 14-d and 28-d isotope incorporation period. We compared (log-transformed) FIA from the different iron-fortified FUF measured 14 d compared with 28 d after the last test FUF consumption using mixed models with formula and time as fixed effect and subject as a random factor. FIA did not differ when measured after 14-d and 28-d of erythrocyte incorporation (Time, *P* = 0.368; FUF, *P* = 0.435; Time × FUF, *P* = 0.937). The boxes show the medians with 25th and 75th percentiles, and the dots represent individual values. FeSO_4_, ferrous sulfate; 2′FL, 2′-fucosyllactose.

In the subsample of 26 children, FIA from the 3 isotopically labeled FUF did not differ when measured after 14-d and 28-d of erythrocyte incorporation (time, *P* = 0.368; FUF, *P* = 0.435; time × FUF, *P* = 0.937) (Figure 3).

### Factors associated with iron absorption

Multiple linear regression models were run to explore the factors associated with FIA from the different test FUF (Table 2). Hb was negatively associated with FIA from the control FUF [β: −0.42 (−0.75, 0.09)] and FUF with 2′FL [β: −0.37 (−0.72, 0.02)], but did not reach significance for FUF with synbiotic [β: −0.29 (−0.66, 0.09), *P* = 0.130]. Fecal pH was negatively associated with FIA from all 3 test FUF [control, β: −7.93 (−11.5, −4.36); synbiotic, −6.11 (−10.11, −2.11); 2′FL, −6.89 (−10.66, −3.11)].

**TABLE 2:** Multiple linear regression models investigating fractional iron absorption (%) from iron-fortified follow-up formula (FUF) with and without the addition of synbiotic or 2′-fucosyllactose in 8–14 months-old Thai children.

Follow-up formula	Variable	β	95% CI	Std β	*P* value
	Hemoglobin (g/L)	−0.42	−0.75, −0.09	−0.28	0.014
Control (*n* = 68)	Plasma ferritin (μg/L)	−0.11	−0.24, 0.02	−0.18	0.093
Adjusted *R*^2^ = 0.38	Soluble transferrin receptor (mg/L)	−0.04	−0.55, 0.05	−0.02	0.884
	CRP (mg/L)	−0.13	−0.98, 0.71	−0.031	0.760
	Age (week)	−0.11	−0.56, 0.35	−0.049	0.647
	Fecal pH	−7.92	−11.50, −4.36	−0.05	<0.001
	Hemoglobin (g/L)	−0.29	−0.66, 0.09	−0.19	0.130
	Plasma ferritin (μg/L)	−0.10	−0.24, 0.05	−0.15	0.201
Synbiotic (*n* = 68)	Soluble transferrin receptor (mg/L)	0.04	−0.53, 0.62	0.02	0.879
Adjusted *R*^2^ = 0.20	C-reactive protein (mg/L)	−0.11	−1.05, 0.08	−0.03	0.819
	Age (week)	0.04	−0.47, 0.55	0.02	0.870
	Fecal pH	−6.11	−10.11, −2.11	−0.37	0.003
	Hemoglobin (g/L)	−0.37	−0.72, −0.02	−0.24	0.039
	Plasma ferritin (μg/L)	−0.13	−0.27, 0.01	−0.21	0.059
2'-fucosyllactose (*n* = 68)	Soluble transferrin receptor (mg/L)	0.07	−0.47, 0.61	0.03	0.787
Adjusted *R*^2^ = 0.31	C-reactive protein (mg/L)	−0.15	−1.05, 0.74	−0.04	0.731
	Age (week)	−0.04	−0.52, 0.45	−0.02	0.885
	Fecal pH	−6.89	−10.66, −3.11	−0.41	0.001

The estimated β, 95% CI, and std. β values for fractional iron absorption from the test formulas were assessed by multiple linear regression models. Predictors were assessed from variables at baseline, *n* = 68. Follow-up formulas contained 0.5 mg native iron and 2.20 mg isotopically labeled iron and *1*) synbiotic [(GOS) + *L. reuteri*], *2*) 2′-fucosyllactose (2′FL), or *3*) without synbiotic or 2′FL (control).

Abbreviations: CI, confidence interval; GOS, galacto-oligosaccharide.

We explored whether PF <12 μg/L, Hb <110 g/L, or fecal pH above or below the median modified the effect of synbiotic or 2′FL on FIA but found no interaction of these variables with synbiotic or 2′FL on FIA in the linear mixed models with formula as fixed factor and subject as random factor (data not shown).

## Discussion

The main findings of this stable iron isotope study in 8–14 mo-old Thai children consuming single servings of FUF fortified with iron as FeSO_4_ are that *1*) the addition of either a synbiotic (GOS + *L.reuteri*) or 2′FL did not affect iron absorption; *2*) FIA from the 3 different test FUF did not differ when measuring erythrocyte incorporation of isotopic labels 14 d compared with 28 d after administration; and *3*) Hb and fecal pH were negatively associated with FIA. Our findings do not support the hypothesis that supplementation of FUF with synbiotics or 2′FL modifies iron absorption in infants and young children. These findings differ from previous stable iron isotope studies showing an increase in iron absorption in infants and female adults consuming prebiotics [8,35,36] and in female adults consuming probiotics [12,13]. As discussed below, potential reasons for this disparity include differences in iron compound, presence of inhibitors and enhancers in the test meal, prebiotic compound and probiotic strain, dose and/or exposure duration, and/or subjects’ iron status.

Previous studies showed that GOS enhances iron absorption from ferrous fumarate (FeFum) and sodium iron ethylenediaminetetraacetic acid (NaFeEDTA), but not from FeSO_4_ [8,35,37]. Anemic Kenyan infants (aged 6–14 mo) consumed maize porridge fortified with a micronutrient powder containing 5 mg iron with 7.5 g of GOS or without GOS for 3 wk [8]. GOS consumption significantly increased iron absorption by 62% from FeFum + NaFeEDTA but not from FeSO_4_ [8]. In contrast, a follow-up study in mainly iron deficient and anemic Kenyan infants by Mikulic et al. [37] reported no increase in iron absorption when GOS (7.5 g) was added to a single serving of maize porridge fortified with 5 mg iron as FeFum + NaFeEDTA or FeSO_4_. In iron-depleted Swiss females, 15 g of GOS significantly increased FIA from FeFum (14 mg iron) given with water (+61%) or with a noninhibitory test meal (+28%), but did not enhance iron absorption from FeSO_4_ [35,36]. Thus, the results from our study agree with previous stable iron isotope studies showing no enhancing effect of GOS on iron absorption from FeSO_4_ or when GOS was administered as a single dose.

The lack of impact of GOS on iron absorption from FeSO_4_ may be due to its physicochemical characteristics. It is readily soluble in water and gastric juice and is used as a reference compound for iron absorption [38]. In contrast, iron compounds that are poorly soluble in water and soluble in diluted acids, such as FeFum [39], would need a highly acidic gastric pH ∼2 or longer time for a full dissolution [40]. These differences may partly explain the impact of GOS on FeFum, which appears to increase its solubility at physiologic pH [41], whereas no effect is found on FeSO_4_ which is already highly soluble and readily absorbable, especially in the presence of ascorbic acid.

Because of its relatively high bioavailability, FeSO_4_ is the iron compound of choice for iron fortification of infant formula and FUF [23]. Furthermore, according to Codex Alimentarius, all infant formula and FUF must be fortified with ascorbic acid [42], which is the most potent enhancer of nonheme iron absorption [43]. In our study, the median FIA from FeSO_4_ in all 3 test FUF was ∼ 8.5 %, which is similar to the median FIA values from FeSO_4_-fortified formula reported in previous iron absorption studies in infants (2–8 mo old) ranging from 7% to 11% [24,[44], [45], [46]]. In our study, 1 serving of FUF provided 36 mg ascorbic acid in addition to 2.25 mg iron (molar ratio of iron to ascorbic acid: 1:5.1). Ascorbic acid has been reported to enhance iron absorption in FeSO_4_-fortified formula from a minimum iron to ascorbic acid molar ratio of 1:2 [47]. Thus, it is likely that the addition of GOS cannot further enhance the absorption of FeSO_4_ in the presence of ascorbic acid.

In addition, previous studies were carried out in mainly iron-depleted and/or anemic subjects. In the current study, iron depletion and/or anemia were not an inclusion criterion, and only 21% and 15% of children were iron deficient and anemic, respectively. Hb was negatively associated with FIA (significance only reached for control FUF and FUF with 2′FL). Thus, an enhancing effect of GOS may only be achieved in anemic children with upregulated iron absorption. We did not find anemia to be an effect modifier, but the number of anemic children in the sample was likely too small to detect a significant anemia-FUF interaction.

Another reason for not observing an enhancing effect of GOS on iron absorption in the current study could be attributed to the lower dose of GOS administered compared with previous studies. The enhancing effect of GOS on iron absorption from FeFum appears to be dose-dependent. In iron-deplete females, a single dose of GOS enhanced FIA from 14 mg iron at a dose of 15 and 7 g, but not at 3.5 g [35,36]. In Kenyan infants, GOS improved FIA from 5 mg iron at a dose of 7.5 g [8,37], which is 8-fold higher than the amount of GOS provided with our test FUF (0.94 g/235 mL serving or 0.4 g/100 mL). However, the amount of GOS and fructo-oligosaccharides that can be added to FUF is set by EU regulation (2016/127) and may not exceed 0.8 g/100 mL.

Furthermore, the findings of previous studies in Kenyan infants indicate that GOS may increase iron absorption (from FeFum + NaFeEDTA) after repeated exposure but not from a single dose [37]. It has been proposed that GOS enhances colonic iron absorption by reducing pH through bacterial fermentation, which would likely require chronic exposure [8]. Studies indicate that fecal pH is lower in breastfed infants and infants fed with GOS-containing formula compared with those fed infant formula without added prebiotics [48]. Notably, we observed that a lower fecal pH was associated with higher FIA, similar to findings from previous studies [8,37]. Although it is uncertain whether lower fecal pH represents lower pH in the proximal colon, lower colonic pH may increase the solubility of minerals in the colonic lumen. Lower colonic pH has been associated with higher calcium and magnesium absorption [45,46], thus, may also improve colonic iron absorption through this same mechanism.

To our knowledge, this is the first stable iron isotope study to determine the possible effects of an HiMO on iron absorption from iron-fortified formula. Our finding agrees with a recent study in partially breastfed Kenyan infants that found no effect of 2′FL (2 g) and lacto-N-neotetraose (1 g) on iron absorption from iron-fortified (5.0 mg as FeFum) maize porridge, whereas the addition of GOS (3 g) increased FIA by 78% [22]. HiMO are complex glycans composed of 5 different monosaccharides, in either branched or linear forms [49]. In contrast, GOS is linear, consisting of 2 monosaccharides [17]. Reducing sugars of dietary fibers can form stable soluble complexes with iron [50,51]. However, the effects may vary across different fibers and remain poorly understood [52].

Most previous iron absorption studies in infants and young children determined FIA by measuring the incorporation of isotopic labels 14 d after the last test meal administration [53]. Fomon et al. [24] investigated the time course of iron isotope incorporation into erythrocytes from iron-fortified (^58^FeSO_4_) formula in infants aged 2 and 5 mo. Erythrocyte incorporation of the iron isotope significantly increased beyond 14 d after administration and plateaued around 28 d in 2-mo-old infants after consuming both a low iron (2 mg/L) and high iron (12 mg/L) formula. The iron incorporation patterns were similar in 5-mo-old infants, but incorporation increased significantly beyond 14 d only after consumption of the high iron formula. In contrast, we did not find significant nor relevant differences in FIA when measured after a 14- and 28-d incorporation period in our participants aged between 8 and 14 mo. Similarly, an earlier study by Fomon et al. [54] reported no difference in FIA from ^58^Fe-fortified formula (1.8 mg/L) when measured 14 and 42 d after consumption in 2-mo-old infants. Our results confirm that at low iron dose, iron incorporation into erythrocytes may plateau after a 14-d period in older infants and young children, similar to adults.

Strengths of our study include the assessment of iron absorption using multiple stable iron isotope labels (^54^Fe, ^57^Fe, and ^58^Fe) allowing within-subject comparisons within 1 wk of test FUF administrations, which may reduce variability. Participants were randomly assigned to the FUF administration sequence with 3 d of washout between each test FUF administration. This likely diminished the potential carry-over effects of the previous FUF as we do not expect a single administration to have a lasting impact on the gut microbiome and/or colonic pH, which was confirmed by a lack of differences in FIA by administration sequence. Furthermore, our study had a relatively large sample size for a stable iron isotope absorption study. It was, however, not a priori powered to compare FIA from FUF with added synbiotic to FIA from FUF with added 2′FL due to limited evidence pointing toward a potential superiority of the synbiotic or 2′FL in enhancing iron absorption.

Limitations of our study include the low dose of GOS compared with previous iron isotope studies, the inclusion of mostly nonanemic and iron-sufficient children, and the pre- and probiotic being tested in a formulation where iron is already highly bioavailable. Furthermore, our participating children had been previously exposed to pre- and/or probiotic-containing infant formula or FUF, which may have unknown effects on the gut microbiome and iron absorption. However, we provided all children with a FUF without pre- or probiotics during a 14-d run-in period and the week of test FUF administration, minimizing any residual effects of previous consumption. We did not determine the effect of the synbiotic or 2′FL on FIA after chronic consumption, only from a single serving. In our single-blinded study, participants were blinded to the 3 trial arms (test FUF), but the test FUF administrators were not. The independent statistician conducting the statistical analyses was blinded. Furthermore, as the primary outcome (FIA) is measured in the same blood sample and cannot be influenced by the participant or researcher, we believe that the lack of double-blinding did not present a relevant source of bias. We recognize that our study did not fully adhere to the guidance for the conduct and reporting of clinical trials of breast milk substitutes by Jarrold et al. [55]. The reason is that this guidance had not yet been published when our study was designed. Specifically, our study protocol did not state how partially breastfeeding females were supported during the study, and participants received free feeding equipment and FUF during the study, which could have been seen as an incentive to use commercial milk formula to be eligible for participation in the study. Although we did not provide an incentive to the partially breastfeeding females to continue breastfeeding during the study, we did encourage females to continue breastfeeding. However, 1 mother stopped breastfeeding during the study.

In conclusion, in mainly iron-sufficient, 8–14 mo-old Thai children with previous exposure to pre- and/or probiotics, the addition of synbiotic or 2′FL to FeSO_4_-fortified FUF containing iron absorption enhancing ascorbic acid did not significantly increase iron absorption from a single serving. It remains to be investigated whether chronic consumption of formula with added pre- and/or probiotics may enhance iron absorption.

## Acknowledgments

We thank Adam Krzystek, Timo Christ, Aria Minder, and Kashish Mallick at ETH Zurich, Switzerland, and Jürgen Erhardt (Willstaett, Germany) for supporting the laboratory analysis. We also thank Yipu Chen, Jowena Lebumfacil, Elias Mardhy, Shaillay Kumar Dogra, and Dominik Grathwohl from Nestlé, Switzerland for study operational support and Bertrand Bétrisey for the development of *L. reuteri* primers used for qPCR.

## Author contributions

The authors’ responsibilities were as follows – MBZ, JB, MS, PS: designed the study; PS, A-SD, SS, TK: conducted the study; CR, JB, CZ, PS: analyzed the data and performed the statistical analyses; CR, JB, PS, MS, SS, MG-G, MBZ: participated in data interpretation; PS, JB: wrote the first draft of the manuscript; and all authors: read and approved the final manuscript.

## Conflict of interest

MS and MG-G are employees of Société des Produits Nestlé S.A. All other authors report no conflicts of interest.

## Funding

The study was supported by the Société des Produits Nestlé S.A., Vevey, Switzerland, and by a Bridging Grant from the Swiss State Secretariat for Education, Research and Innovation (SERI) (BG 01-092019). Nestlé was involved in the design of the study, production of the study formula, interpretation of results, and review of the manuscript, but not in the conduct of the study, the planning, and conduct of statistical analyses.

## Data availability

Data described in the manuscript, code book, and analytic code will be made available on request pending application and approval.
