# Supplementary material for: Iron Absorption from an Iron-Fortified Follow-Up Formula with and without the Addition of a Synbiotic or a Human-Identical Milk Oligosaccharide: A Randomized Crossover Stable Isotope Study in Young Thai Children
Source: J Nutr. 2024 Aug 22;154(10):2988–98. doi: 10.1016/j.tjnut.2024.08.016 (PMC11522886; doi:10.1016/j.tjnut.2024.08.016)
Supplement: Multimedia component 1 [file mmc1.docx]

**Iron absorption from an iron-fortified follow-up formula with and without the addition of a synbiotic or human milk oligosaccharide: a randomized cross-over stable isotope study in young Thai children**

Scheuchzer et al.

**SUPPLEMENTARY MATERIALS**

**Supplementary Table 1.** Composition of investigational FUF per serving size of 235 mL

| Composition | Wash-out FUF (low in iron and without pre-and probiotics) | Control FUF | FUF + Synbiotic | FUF + 2'FL |
| --- | --- | --- | --- | --- |
| Energy, kcal | 156 | 156 | 156 | 156 |
| Protein, g | 3.46 | 3.46 | 3.46 | 3.46 |
| Total fat, g | 7.11 | 7.24 | 7.24 | 7.24 |
| Carbohydrates, g | 19.6 | 19.3 | 19.3 | 19.3 |
| Native iron (mg) | 0.05 | 0.05 | 0.05 | 0.05 |
| Added iron as FeSO_4_ (mg) | 1.85 | na | na | na |
| Added iron as isotopic iron, FeSO_4_ (mg) | na | 2.20 as ^58^Fe | 2.20 as ^57^Fe | 2.20 as ^54^Fe |
| Zinc (mg) | 1.2 | 1.36 | 1.36 | 1.36 |
| Calcium (mg) | 233 | 233 | 233 | 233 |
| Vitamin A (µgRE) | 200 | 200 | 200 | 200 |
| Vitamin C (mg) | 36 | 36 | 36 | 36 |
| GOS (mg) | na | na | 940 | na |
| 2'FL (mg) | na | na | na | 235 |
| *L. reuteri* (CFU) | na | na | 6.4*10^6^ | na |

Abbreviations: 2'FL, 2'-fucosyllactose; Fe, iron; FUF, follow-up formula; GOS, galacto-oligosaccharide; *L. reuteri*, *Limosilactobacillus reuteri* DSM 17938; na, not applicable.

**Supplementary Table 2.** Baseline characteristics of the 8–14 months-old Thai children (per-protocol sample)

| Characteristic | n | Value |
| --- | --- | --- |
| Age, weeks | 63 | 51.4 ± 7.1^1^ |
| Male/female, n (%) | 63 | 28/35 (44/56) |
| Exclusively breastfed, n (%) | 63 | 23 (30) |
| Introduction to formula milk, month | 63 | 3 (1, 6)^2^ |
| Receiving human milk during the study, n (%) | 63 | 14 (22) |
|  |  |  |
| *Anthropometrics* |  |  |
| Weight, kg | 63 | 9.3 ± 1.2 |
| Length, cm | 63 | 74.3 ± 2.9 |
| Weight-for-age z-score | 63 | -0.1 ± 1.0 |
| Weight-for-length z-score | 63 | 0.0 ± 1.0 |
| Length-for-age z-score | 63 | -0.1 ± 1.0 |
|  |  |  |
| *Iron status* |  |  |
| Hemoglobin, g/L | 62 | 121 ± 9.7 |
| Anemia, n (%)^3^ | 62 | 9 (14.5) |
| Plasma ferritin, µg/L | 58 | 30.3 (20.0, 46.8) |
| Plasma ferritin adjusted^4^, µg/L | 58 | 26.8 (18.7, 45.7) |
| <12 µg/L, n (%) | 58 | 6 (10) |
| Soluble transferrin receptor, mg/L | 58 | 6.3 (5.3, 7.3) |
| >8.3mg/L, n (%) | 58 | 10 (17) |
| Body iron stores (mg/kg body weight) | 58 | 4.4 (2.5, 5.7) |
| Iron deficiency, n (%)^5^ | 58 | 14 (24) |
| Iron deficiency anemia^6^, n (%) | 58 | 4 (7) |
|  |  |  |
| *Systemic inflammation* |  |  |
| C-reactive protein, mg/L | 58 | 0.1 (0.0, 0.7) |
| > 5 mg/L, n (%) | 58 | 0 (0) |
| α-1-acid glycoprotein, g/L | 58 | 0.6 (0.5, 0.8) |
| > 1 mg/L, n (%) | 58 | 5 (7) |
|  |  |  |

^1^Mean (±SD), all such values.

^2^Median (IQR), all such values.

^3^Anemia defined as Hb <110 g/L.

^4^Plasma ferritin adjusted for inflammation by BRINDA.

^5^Iron deficiency was defined as adjusted PF <12 μg/L and/or elevated sTfR >8.3 mg/L.

^6^Iron deficiency anemia defines as the combination of anemia and iron deficiency.
